# Factors Associated With Rectal Spacer Use in Prostate Cancer Patients Receiving Radiation Therapy

**DOI:** 10.1002/cam4.71765

**Published:** 2026-04-28

**Authors:** Ryan A. Hankins, Kathryn C. Morris, Alysha M. McGovern, Jennifer Zack, Sean P. Collins, James B. Yu

**Affiliations:** ^1^ MedStar Georgetown University Hospital Washington DC USA; ^2^ Boston Scientific Marlborough Massachusetts USA; ^3^ Georgetown University School of Medicine Washington DC USA; ^4^ USF Health Morsani College of Medicine Tampa Florida USA; ^5^ Dartmouth Hitchcock Medical Center Lebanon New Hampshire USA; ^6^ Cancer Outcomes, Public Policy, and Effectiveness Research (COPPER) Center at Yale New Haven Connecticut USA

**Keywords:** disparities, prostate cancer, proton therapy, radiation therapy, rectal spacer, rectal toxicity, SBRT, utilization

## Abstract

**Background/Objective:**

Prostate cancer is a leading cause of cancer‐related death among men in the United States. However, the influence of demographic, socioeconomic, regional, and treatment‐related factors on rectal spacer use among men treated with radiation therapy remains largely unexplored in the literature. This study evaluated rectal spacer utilization among Medicare beneficiaries with prostate cancer by radiation therapy modality, region, race, income, and year.

**Methods:**

The Medicare 5% Standard Analytical Files were used to identify men aged 65+ with prostate cancer who underwent treatment with intensity‐modulated radiotherapy (IMRT), stereotactic body radiation (SBRT), brachytherapy, or proton therapy from 1/1/2017–12/31/2021. Patients were stratified by rectal spacer placement within 60 days pre‐radiation therapy initiation.

**Results:**

A total of 66,680 patients were identified (mean age 72.8 years, mean Charlson Comorbidity Index score 3.26, 80.7% White). Among this cohort, 17,940 patients (26.9%) received a rectal spacer. Spacer utilization increased significantly over time, from 6.8% in 2017 to 42.4% in 2021 (*p* < 0.05). Geographic variation was observed, with the highest utilization in the West (31.7%) and the lowest in the Northeast (23.6%). A greater proportion of White patients received rectal spacers than Black patients in all regions except the Northeast (West: 32.6% vs. 22.2%; South: 30.8% vs. 20.5%; Midwest: 24.4% vs. 15.5%; all *p* < 0.001), where the opposite was observed (31.8% vs. 22.2%; *p* < 0.001). Rectal spacer utilization increased with income, ranging from 20.9% in the lowest income quintile to 28.2% in the highest (*p* < 0.001). By treatment modality, utilization was highest among patients receiving proton therapy (59.0%), followed by SBRT (46.0%), brachytherapy (23.7%), and IMRT (20.6%).

**Conclusions:**

Rectal spacer utilization increased significantly from 2017 to 2021, with the greatest relative increases among proton therapy patients and SBRT patients. Significant variations based on geographic region, race, and income highlight the complex interplay of these factors on treatment access and decision‐making.

## 1 Background

The effectiveness of radiation therapy in treating prostate cancer depends on several factors, including the ability to deliver an adequate radiation dose to the prostate while minimizing exposure to nearby healthy tissue, particularly the rectum. While definitive radiation therapy is associated with positive disease‐free survival rates [1], treatment‐related morbidity can significantly impact patients' quality of life (QoL) [2] and may limit the tumor‐controlling radiation dose that can be administered [3]. Newer radiation therapy techniques, such as image‐guided radiation therapy, have improved the precision of radiation delivery to the prostate [3]. However, doses to the rectum may remain high [4, 5, 6], and the proximity of surrounding healthy tissues or adjacent ‘organs‐at‐risk’ (OARs) may limit further dose escalation [7].

Radiation toxicity symptoms may begin during treatment, but often do not appear until months or even years later [8, 9, 10, 11, 12]. Gastrointestinal symptoms, such as diarrhea, rectal bleeding, painful defecation, and fecal urgency, may occur [2]. In rare cases, severe complications like rectal fistulas may necessitate surgery and hospitalization [13, 14]. These debilitating side effects of rectal toxicity can persist for up to a decade or longer [15], severely reducing the QoL of prostate cancer patients [2]. Rectal spacers have emerged as an effective solution to reduce radiation dose to the rectum, physically displacing the rectum and enabling safer delivery of higher radiation doses [7, 16, 17, 18, 19]. Several types of rectal spacers have been used for this purpose, including absorbable hydrogel spacers, hyaluronic acid, collagen, and implantable absorbable balloons [20].

To date, no previously published studies have reported utilization data for rectal spacers in prostate cancer patients undergoing radiation therapy. A better understanding of rectal spacer utilization among United States (US) Medicare beneficiaries may help providers and healthcare payers identify opportunities to enhance the quality of care for prostate cancer patients while simultaneously reducing healthcare costs. This study aimed to evaluate rectal spacer use in US Medicare patients with prostate cancer, analyzing patterns by radiation therapy modality, geographic region, race, and income.

## 2 Methods

### 2.1 Study Design

The intent of the study was to conduct a descriptive analysis of real‐world trends in rectal spacer utilization among Medicare beneficiaries, focusing on temporal trends and variations by demographic, regional, and socioeconomic patterns. Rather than modeling predictors or outcomes, this study characterized population‐level adoption patterns across key demographic and treatment subgroups, particularly as they pertain to disparities in advanced radiation delivery techniques.

### 2.2 Data Source

This retrospective claims‐based analysis utilized the Medicare 5% Standard Analytical Files (SAF), which contain a 5% random sample of traditional Medicare fee‐for‐service beneficiaries, the majority of whom are aged 65 and older. The database provides comprehensive claims data for Medicare Part A and Part B services. Because Medicare beneficiaries typically remain enrolled until death, the SAF database supports robust longitudinal analysis.

The study was determined to be exempt from Institutional Review Board (IRB) approval as this research project did not involve human subjects and used data from an anonymous, de‐identified medical clearinghouse of administrative claims compliant with the Health Insurance Portability and Accountability Act of 1996.

### 2.3 Patient Population

The Medicare 5% SAF were used to identify men aged 65 and older who were diagnosed with prostate cancer between January 1, 2017 and December 31, 2021, and subsequently treated with radiation therapy. Patients who received intensity‐modulated radiotherapy (IMRT), stereotactic body radiation therapy (SBRT), brachytherapy, or proton therapy following their prostate cancer diagnosis were included (see Table 1 for codes used to identify radiation therapy). Eligible patients were required to have continuous Medicare fee‐for‐service enrollment for at least 3 years prior to and at least 1 year following their index radiation therapy treatment date to allow for identification of exclusion criteria. Men who had undergone a radical prostatectomy during the three‐year pre‐index period or who were diagnosed with any non‐prostate cancer malignant neoplasm at any time during the study period were excluded.

**TABLE 1:** Codes used to identify radiation therapy.

CPT code	Code description
IMRT	
77,385	Intensity modulated radiation treatment delivery (IMRT), includes guidance and tracking, when performed; simple
77,386	Intensity modulated radiation treatment delivery (IMRT), includes guidance and tracking, when performed; complex
SBRT	
77,373	Stereotactic body radiation therapy, treatment delivery, per fraction to 1 or more lesions, including image guidance, entire course not to exceed 5 fractions
Brachytherapy	
0395 T	High dose rate electronic brachytherapy, interstitial or intracavitary treatment, per fraction, includes basic dosimetry, when performed
77,761	Intracavitary radiation source application; simple
77,762	Intracavitary radiation source application; intermediate
77,763	Intracavitary radiation source application; complex
77,768	Remote afterloading high dose rate radionuclide skin surface brachytherapy, includes basic dosimetry, when performed; lesion diameter over 2.0 cm and 2 or more channels, or multiple lesions
77,770	Remote afterloading high dose rate radionuclide interstitial or intracavitary brachytherapy, includes basic dosimetry, when performed; 1 channel
77,771	Remote afterloading high dose rate radionuclide interstitial or intracavitary brachytherapy, includes basic dosimetry, when performed; 2–12 channels
77,772	Remote afterloading high dose rate radionuclide interstitial or intracavitary brachytherapy, includes basic dosimetry, when performed; over 12 channels
77,778	Interstitial radiation source application, complex, includes supervision, handling, loading of radiation source, when performed
77,790	Supervision, handling, loading of radiation source
Proton Therapy	
77,520	Proton treatment delivery; simple, without compensation
77,522	Proton treatment delivery; simple, with compensation
77,523	Proton treatment delivery; intermediate
77,525	Proton treatment delivery; complex

*Note:* CPT is a registered trademark of the American Medical Association.

Abbreviations: CPT, Current Procedural Terminology (CPT) codeIMRT, intensity‐modulated radiotherapy; SBRT, stereotactic body radiation therapy.

### 2.4 Study Measures

Eligible patients were stratified based on whether they received a rectal spacer, identified by the presence of Current Procedural Terminology Code (CPT) code 55,874 or 0438 T, within 60 days prior to the start date of their radiation therapy. The annual proportions of men receiving a rectal spacer from 2017 through 2021 were analyzed. Baseline demographic characteristics such as age, race, and Census region (Northeast, Midwest, West, or South) were evaluated and compared between patients with and without a spacer. Additionally, county‐level 2022 median household income (MHI) data from the US Census Bureau Small Area Income and Poverty Estimates dataset were cross‐referenced with patient county codes to categorize patients into income quintiles (Q1: ≤ $50,533, Q2: $50,534–$57,495, Q3: $57,496–$64,037, Q4: $64,038–$73,429, Q5: ≥ $73,430). This classification allows for an examination of socioeconomic factors in relation to treatment outcomes. Also, the annual proportions of rectal spacer utilization were calculated for each radiation therapy modality, and changes over time were described using absolute percentage point differences.

### 2.5 Statistical Analyses

Continuous variables were reported as means with standard deviations (SD), while categorical variables were summarized as frequencies and percentages. Per the cell size suppression policy of the Centers for Medicare and Medicaid (CMS) Data User Agreement (DUA), all cell counts less than 11 have been suppressed in this document, tables, and figures to protect patient confidentiality. Also, percentages or other calculations that could allow derivation of suppressed cell counts (< 11) were not performed or reported. Where appropriate, proportions or aggregated categories were used in place of exact patient counts.

To estimate nationwide Medicare patient numbers, counts from the 5% Medicare SAF sample were multiplied by 20, extrapolating to the total Medicare population. Differences in categorical characteristics between 2017 and 2021 were assessed using a chi‐square test. All analyses were performed using the Instant Health Data (IHD) software (Panalgo, Boston MA, USA) and R, version 3.2.1 (R Foundation for Statistical Computing, Vienna, Austria).

## 3 Results

### 3.1 Patient Demographic and Clinical Characteristics

A total of 66,680 prostate cancer patients who received radiation therapy were identified. The mean age of patients was 72.8 years (SD = 4.6), and the mean Charlson Comorbidity Index (CCI) score was 3.26 (SD = 1.59), indicating a moderate burden of comorbid conditions (Tables 2, 3, 4). The majority of patients were White (80.7%), and most resided in the Southern (38.9%) or Midwest (23.0%) regions of the US. Nearly half of the patients (48.6%) were in the highest income quintile, with a county‐level MHI ≥ $73,430.

**TABLE 2:** Demographic and clinical characteristics of US Medicare patients with prostate cancer and any radiation therapy type.

	Any radiation therapy type			
	Overall (*n* = 66,680)	Rectal spacer (*N* = 17,940)	No rectal spacer (*n* = 48,740)	*p*
Age				
Mean (SD)	72.8 (4.6)	72.8 (4.6)	72.8 (4.6)	0.9012
Age group				
65–74	67.8%	68.0%	67.7%	0.8854
75+	32.2%	32.0%	32.3%	
Race				
White	80.7%	82.7%	79.9%	0.0211
Black	12.1%	9.7%	12.9%	
Other	4.2%	3.7%	4.4%	
Unknown	3.1%	3.9%	2.8%	
CCI score				
Mean (SD)	3.26 (1.59)	3.23 (1.56)	3.27 (1.60)	0.5217
CCI score grouping				
0–1	0.4%	0%	0.6%	
2	44.6%	45.9%	44.2%	
3+	55.0%	54.1%	55.3%	
Census region				
Midwest	23.0%	20.6%	23.9%	0.0017
Northeast	22.0%	19.2%	23.0%	
South	38.9%	41.2%	38.0%	
West	16.2%	19.0%	15.1%	
MHI				
Mean (SD)	$76,974	$78,505	$76,410	0.0142
MHI Quintile (Q)				
Q1 (≤$50,533)	5.4%	4.2%	5.9%	0.1757
Q2 ($50,534–$57,495)	10.3%	9.8%	10.5%	
Q3 ($57,496–$64,037)	16.3%	15.0%	16.8%	
Q4 ($64,038–$73,429)	19.4%	20.2%	19.1%	
Q5 (≥$73,430)	48.6%	50.8%	47.8%	

Abbreviations: CCI, Charlson Comorbidity Index; CMS, Centers for Medicare & Medicaid Services; MHI, median household income; SD, standard deviation; US, United States.

**TABLE 3:** Demographic and clinical characteristics of US Medicare patients with prostate cancer by radiation therapy type (Brachytherapy and IMRT).

	Brachytherapy				IMRT			
	Overall (*n* = 12,980)	Rectal spacer (*n* = 3080)	No rectal spacer (*n* = 9900)	*p*	Overall (*n* = 45,280)	Rectal spacer (*n* = 9340)	No rectal spacer (*n* = 35,940)	*p*
Age								
Mean (SD)	72.0 (4.1)	72.3 (4.2)	71.9 (4.1)	0.2884	73.1 (4.7)	73.2 (4.7)	73.0 (4.7)	0.519
Age Group								
65–74	74.4%	69.5%	76.0%	0.1327	64.8%	63.6%	65.1%	0.5791
75+	25.6%	30.5%	24.0%		35.2%	36.4%	34.9%	
Race								
White	80.9%	88.3%	79.9%	^a^	80.2%	81.2%	79.9%	0.2968
Black	11.7%	^a^	^a^		12.6%	12.6%	12.6%	
Other	4.8%	^a^	^a^		4.3%	2.8%	4.7%	
Unknown	2.6%	^a^	^a^		2.9%	3.4%	2.8%	
CCI score								
Mean (SD)	3.12 (1.48)	3.09 (1.52)	3.14 (1.47)	0.7495	3.33 (1.64)	3.43 (1.70)	3.31 (1.62)	0.1466
Census region								
Midwest	19.9%	13.1%	22.0%	< 0.0001	25.6%	23.3%	26.1%	0.045
Northeast	12.4%	10.5%	13.0%		24.9%	21.6%	25.7%	
South	45.3%	32.7%	49.3%		35.1%	40.3%	33.8%	
West	22.4%	43.8%	15.7%		14.4%	14.8%	14.4%	
MHI								
Mean (SD)	$79,295	$83,200	$78,087	0.0251	$76,017	$77,438	$75,647	0.1153

Abbreviations: CCI, Charlson Comorbidity Index; CMS, Centers for Medicare & Medicaid Services; IMRT, intensity‐modulated radiotherapy; MHI, median household income; SD, standard deviation; US, United States.

^a^ Per CMS cell suppression policy, values of < 11 have been suppressed to protect patient confidentiality.

**TABLE 4:** Demographic and clinical characteristics of US Medicare patients with prostate cancer by radiation therapy type (Proton Therapy and SBRT).

	Proton therapy				SBRT			
	Overall (*n* = 5120)	Rectal spacer (*n* = 3020)	No Rectal spacer (*n* = 2100)	*p*	Overall (*n* = 7560)	Rectal spacer (*n* = 3480)	No Rectal spacer (*n* = 4080)	*p*
Age								
Mean (SD)	72.2 (4.6)	71.9 (4.3)	72.5 (5.0)	0.3322	72.6 (4.3)	72.8 (4.5)	72.4 (4.1)	0.2985
Age group								
65–74	74.2%	75.5%	72.4%	0.6779	69.6%	69.0%	70.1%	0.8994
75+	25.8%	24.5%	27.6%		30.4%	31.0%	29.9%	
Race								
White	84.8%	84.1%	85.7%	^a^	79.1%	79.9%	78.4%	^a^
Black	7.8%	^a^	^a^		11.6%	11.5%	11.8%	
Other	^a^	^a^	^a^		4.2%	^a^	^a^	
Unknown	^a^	^a^	^a^		5.0%	^a^	^a^	
CCI score								
Mean (SD)	2.88 (1.28)	2.91 (1.27)	2.85 (1.31)	0.7163	3.28 (1.58)	3.19 (1.48)	3.36 (1.65)	0.298
Census region								
Midwest	10.2%	10.7%	^a^	^a^	20.1%	25.9%	15.2%	0.0462
Northeast	7.5%	8.7%	^a^		27.5%	28.2%	27.0%	
South	59.2%	62.0%	55.2%		38.6%	33.3%	43.1%	
West	23.1%	18.7%	29.5%		13.8%	12.6%	14.7%	
MHI								
Mean (SD)	$77,098	$76,540	$77,897	0.5778	$81,542	$80,411	$82,488	0.4016

Abbreviations: CCI, Charlson Comorbidity Index; CMS, Centers for Medicare & Medicaid Services; MHI, median household income; SBRT, stereotactic body radiation therapy; SD, standard deviation; US, United States.

^a^ Per CMS cell suppression policy, values of < 11 have been suppressed to protect patient confidentiality.

### 3.2 Rectal Spacer Utilization by Geographic Region, Income, Race, and Radiation Therapy Treatment Modality

Among the 66,680 prostate cancer patients who received radiation therapy, 17,940 (26.9%) received a rectal spacer. The majority of spacers were placed in an outpatient setting (69.2%) or in a physician's office (25.4%), with only 3.1% inserted in surgical centers. Spacer utilization varied by region, with the highest proportion of patients receiving a spacer in the West (31.7%) and the lowest in the Northeast (23.6%) (Table 5). The states with the highest rates of spacer utilization were Utah (68.2%), Louisiana (51.9%), Oklahoma (50.0%), South Carolina (43.4%), Florida (39.4%), and Tennessee (39.0%). White patients had significantly higher rectal spacer utilization than Black patients in all regions except the Northeast, where Black patients had significantly higher utilization than White counterparts (all *p* < 0.001).

**TABLE 5:** Rectal spacer utilization by geographic region, county‐level median household income, radiation therapy type, and race.

		No rectal spacer	Rectal spacer
US census region and race			
Midwest	White	75.6%	24.4%
	Black	84.5%	15.5%
Northeast	White	77.8%	22.2%
	Black	68.2%	31.8%
South	White	69.2%	30.8%
	Black	79.5%	20.5%
West	White	67.4%	32.6%
	Black	77.8%	22.2%
MHI quintile and race			
Q1 (≤ $50,533)	White	78.8%	21.2%
	Black	80.5%	19.5%
Q2 ($50,534–$57,495)	White	73.3%	26.7%
	Black	78.8%	21.2%
Q3 ($57,496–$64,037)	White	75.8%	24.2%
	Black	73.5%	26.5%
Q4 ($64,038–$73,429)	White	70.1%	29.9%
	Black	82.2%	17.8%
Q5 (≥ $73,430)	White	71.2%	28.8%
	Black	78.6%	21.4%
Radiation therapy type and race			
SBRT	White	53.5%	46.5%
	Black	54.5%	45.5%
IMRT	White	79.1%	20.9%
	Black	79.4%	20.6%
Brachytherapy	White	74.1%	25.9%
	Black	^a^	^a^
Proton Therapy	White	41.5%	58.5%
	Black	^a^	^a^

Abbreviations: CMS, Centers for Medicare & Medicaid Services; IMRT, intensity‐modulated radiotherapy; MHI, Median Household Income; Q1, quintile 1; Q2, quintile 2; Q3, quintile 3; Q4, quintile 4; Q5, quintile 5; SBRT, stereotactic body radiation therapy; US, United States.

^a^ Per CMS cell suppression policy, values of < 11 have been suppressed to protect patient confidentiality.

MHI was significantly associated with rectal spacer utilization, with higher usage observed in wealthier quintiles (20.9% of patients in MHI Q1 vs. 28.2% of patients in MHI Q5; *p* < 0.001) (Table 5). The proportion of Black patients was higher in the lower income quintiles (Q1: 23.2%; Q2: 15.5%) compared to the higher quintiles (Q3: 9.2%; Q4: 11.6%; Q5: 11.5%). White patients had higher rectal spacer utilization than Black patients in the Q2, Q4, and Q5 MHI income quintiles (*p* < 0.001). Consistent with the overall findings, spacer utilization was highest among patients who received proton therapy (White: 58.5%), followed by SBRT (White: 46.5%), brachytherapy (White: 25.9%), and IMRT (White: 20.9%).

### 3.3 Rectal Spacer Utilization Over Time

**FIGURE 1:**
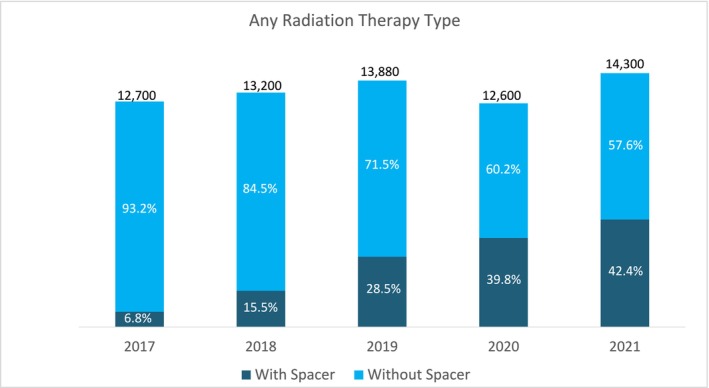
Radiation Therapy and Rectal Spacer Utilization from 2017 to 2021.

The proportion of radiation therapy patients who received a rectal spacer increased significantly from 6.8% in 2017 to 42.4% in 2021 (*p* < 0.05; Figure 1).

### 3.4 Rectal Spacer Utilization by Radiation Therapy Modality and Year

**FIGURE 2:**
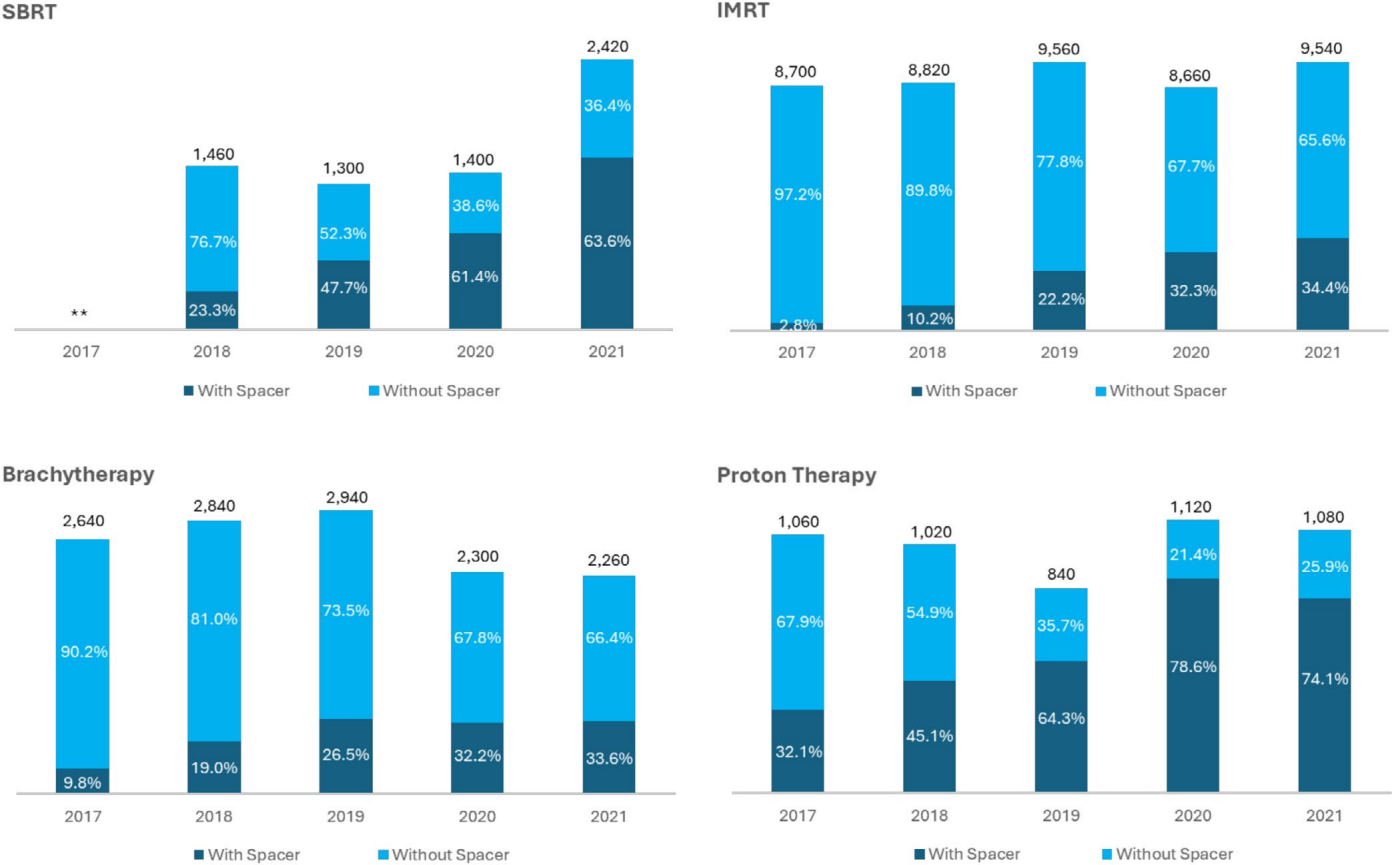
Rectal Spacer Utilization by Radiation Therapy Treatment Modality from 2017 to 2021. *The figure displays absolute annual proportions and the total number of cases by year. Relative or statistical trend analyses were not used in this visual summary. **Per CMS cell suppression policy, values of < 11 have been suppressed to protect patient confidentiality. CMS, Centers for Medicare & Medicaid Services; IMRT, intensity‐modulated radiotherapy; SBRT, stereotactic body radiation therapy.

Between 2017 and 2021, the highest proportion of prostate cancer patients who received rectal spacers was among those treated with proton therapy (59.0%), followed by SBRT (46.0%), brachytherapy (23.7%), and IMRT (20.6%). In 2021, 75.1% of proton therapy patients and 63.6% of SBRT patients received a rectal spacer (Figure 2). Spacer utilization increased across all radiation therapy modalities over time, with the most substantial increase observed in IMRT patients, where usage increased from 2.8% in 2017 to 34.4% in 2021 (Figure 2).

## 4 Discussion

This is the first large observational study to assess the use of rectal spacers in prostate cancer patients undergoing definitive radiation therapy. This analysis of more than 66,000 US Medicare beneficiaries over a five‐year period found 26.9% of patients received a rectal spacer. The use of rectal spacers increased significantly over time, from 6.8% in 2017 to 42.4% in 2021. These findings may highlight a rising demand for rectal spacers, underscoring their potential role in enhancing patient care and minimizing treatment‐related morbidity.

Between 2017 and 2021, spacer utilization was highest among patients receiving proton therapy (59.0%) and patients receiving SBRT (46.0%). In 2021, 75.1% of proton therapy patients and 63.6% of SBRT patients received rectal spacers. Due to the dosimetric properties of proton radiotherapy that may increase rectal toxicity risk [21, 22, 23], including lateral penumbra and end‐range uncertainty, a proportion of patients undergoing proton radiotherapy prior to the development of rectal spacers were treated with daily rectal balloon placement to limit prostate movement and reduce this risk [24]. However, given the discomfort and invasiveness associated with daily rectal balloon use, an increasing number of physicians have transitioned to using rectal spacers when treating patients with proton therapy [25, 26]. However, while this analysis found that spacer utilization was highest among patients receiving proton therapy, recent evidence from the randomized PARTIQoL trial showed no significant differences in efficacy or toxicity between proton therapy and IMRT for low‐ and intermediate‐risk prostate cancer [27, 28]. These results may influence future treatment selection, especially as health systems increasingly emphasize value‐based care. The high cost of proton therapy and spacer placement underscores the need to critically evaluate their additive benefit, particularly in patient populations with low baseline risk of rectal toxicity.

The use of SBRT exhibited the greatest increase over the study period, rising from 1460 patients in 2018 to 2420 patients in 2021, which corresponds to a 65.8% relative increase in utilization. This is a trend consistent with previous research indicating a shift toward shorter radiation therapy courses for prostate cancer between 2004 and 2020 [29]. Growing evidence supports the benefits of rectal spacers in SBRT. Their use has been shown to improve rectoprostatic separation, reduce moderate and high rectal doses when tight planning target volume margins are used, enhance target volume coverage, increase pathological tumor clearance rates, and lower the risk of rectal ulceration [30, 31]. This analysis may highlight a potential trend toward continued expansion of utilization, particularly among patients undergoing ultrahypofractionation and those receiving higher biologically effective doses; however, further research is needed to confirm these patterns [30, 31].

This study also evaluated the impact of socioeconomic factors on rectal spacer utilization in prostate cancer patients. While overall utilization is increasing, significant disparities remain across geographic regions and demographic groups. Spacer utilization was highest among patients in the West and lowest among patients in the Northeast regions of the US. Additionally, White patients were significantly more likely to receive a rectal spacer than Black patients in all regions except the Northeast, where the trend was reversed, with Black patients more likely to receive a spacer. Moreover, patients residing in counties with higher MHI were more likely to receive rectal spacers than patients residing in lower MHI counties. These findings further highlight the complex interplay of socioeconomic, geographic, and racial factors in treatment access.

Although the influence of socioeconomic factors on rectal spacer utilization has not been previously explored in the literature, existing research has documented disparities in prostate cancer incidence, healthcare delivery and access, and clinical outcomes [32, 33]. Studies have shown that Black men have a higher incidence of and mortality from prostate cancer compared to White men [34]. Social determinants of health and racial disparities in treatment were shown to account for most, if not all, of the disparity in prostate cancer mortality [35, 36, 37]. Additionally, Black men were less likely than White men to receive definitive treatment for prostate cancer, particularly for high‐risk disease [38, 39, 40, 41]. However, Black patients (compared with White) and patients who resided in a higher MHI county (compared with lower MHI) have been associated with receiving shorter radiation therapy courses [29]. These findings highlight the need for further research and targeted interventions to address disparities and improve prostate cancer treatment outcomes.

One of the strengths of this study is the large sample size of patients derived from the Medicare database, which allows for robust analysis and comparison. However, it is important to recognize the data used in this study were collected for administrative purposes, rather than specifically for research purposes. Consequently, certain clinical variables may lack detailed information. Potential coding errors and misclassifications within the database could not be identified and, hence, could not be rectified. Claims data may be subject to administrative errors, recording bias, and temporal changes in billing codes [42, 43]. Additionally, the findings from the US Medicare SAF database may not be generalizable to all patients in the US, including commercially insured populations. Because the database primarily includes patients aged 65 and older, the applicability of the results to younger populations who may exhibit different treatment patterns or healthcare access is limited. Furthermore, the analysis is restricted to Medicare fee‐for‐service beneficiaries, excluding patients enrolled in Medicare Advantage plans or other types of private insurance. Finally, while regression analysis could provide additional insights into temporal trends, a stratified descriptive approach was selected for this study as it is more appropriate for identifying practice variation and potential equity gaps at the population level.

Although rectal spacers are generally safe and well tolerated, rare but serious adverse events, including rectal wall infiltration, abscess, rectourethral fistulas, and septic complications, have been reported [44, 45, 46, 47]. In some cases, these events have resulted in colostomy, hospitalization, or death [44, 45]. Also, modern radiotherapy trials with IMRT/SBRT and image guidance demonstrate low rates of delayed Grade 2 and almost no Grade 3 gastrointestinal toxicity [48, 49, 50], and it remains to be seen whether spacer use further improves upon these results and leads to a clinically meaningful benefit. Given this study is a descriptive analysis of utilization patterns, further evaluation of effectiveness and toxicity signals using diagnosis and procedure codes, particularly comparing outcomes between patients with and without rectal spacer placement, represents an important future direction for research. The decision to use a spacer should weigh the procedural risks against the expected benefit in reducing rectal toxicity, particularly in the context of modern radiation therapy techniques that already achieve low toxicity rates [51].

## 5 Conclusions

This study enhances the understanding of patient characteristics and spacer utilization among US Medicare beneficiaries who received radiation therapy for prostate cancer. Rectal spacer utilization significantly increased between 2017 and 2021, with the highest growth observed among patients who received proton therapy or SBRT. However, notable disparities remain, as utilization rates differ significantly across geographic regions and demographic groups. These findings highlight the complex interplay of socioeconomic, geographic, and racial factors influencing treatment access for prostate cancer patients. To address these disparities and enhance treatment equity, further research is essential to identify specific barriers to spacer utilization and to inform targeted interventions aimed at improving patient outcomes.

## Author Contributions

**Ryan A. Hankins:** conceptualization (equal), investigation (equal), methodology (equal), supervision (lead), validation (lead), writing – original draft (equal), writing – review and editing (equal). **Kathryn C. Morris:** conceptualization (equal), formal analysis (supporting), investigation (equal), methodology (equal), writing – original draft (equal), writing – review and editing (equal). **Alysha M. McGovern:** conceptualization (equal), data curation (lead), formal analysis (lead), investigation (equal), methodology (equal), software (lead), writing – original draft (equal), writing – review and editing (equal). **Jennifer Zack:** investigation (equal), writing – original draft (equal), writing – review and editing (equal). **Sean P. Collins:** conceptualization (equal), investigation (equal), methodology (equal), writing – original draft (equal), writing – review and editing (equal). **James B. Yu:** conceptualization (equal), investigation (equal), methodology (equal), writing – original draft (equal), writing – review and editing (equal).

## Funding

This work was supported by Boston Scientific Corporation.

## Conflicts of Interest

The study was supported by Boston Scientific. KCM and AMM are full‐time employees of Boston Scientific. James Yu reports consulting fees from Boston Scientific, Teleflex, and AlphaSights, research support from Pfizer/Myovant/Sumitomo Pharma, and is a stockholder of Modifi Bio. Ryan Hankins is a clinical consultant for Boston Scientific. Sean Collins is a consultant for Boston Scientific.

## Data Availability

This study used administrative claims data from the Centers for Medicare & Medicaid Services (CMS). Due to data use agreements signed with CMS, the data cannot be provided externally. Other researchers can purchase the same dataset to carry out similar analyses.
